# A retrospective cohort analysis comparing the effectiveness and safety of perioperative angiotensin II to adrenergic vasopressors as a first-line vasopressor in kidney transplant recipients

**DOI:** 10.1186/s44158-024-00207-w

**Published:** 2024-10-17

**Authors:** George Urias, Jamie Benken, Hokuto Nishioka, Enrico Benedetti, Scott T. Benken

**Affiliations:** 1 University of Florida Shands Hospital, Gainesville, USA; 2 University of Illinois Chicago College of Pharmacy, Chicago, USA; 3 Department of Medicine, Division of Clinical Anesthesiology, University of Illinois Chicago College of Medicine, Chicago, USA; 4 Department of Surgery, Division of Transplantation , University of Illinois Chicago College of Medicine, Chicago, USA; 5 University of Illinois Chicago College of Pharmacy, Chicago, USA

**Keywords:** Kidney Transplant, Angiotensin II, Dopamine, Phenylephrine, Adrenergic Vasopressors, Catecholamine Vasopressors, Arrhythmias, Ischemia

## Abstract

**Background:**

Perioperative adrenergic vasopressors in kidney transplantation have been linked to negative outcomes and arrhythmias. Synthetic angiotensin II (AT2S) could improve renal hemodynamics, preserve allograft function, and reduce arrhythmias.

**Objective:**

We aimed to compare the effectiveness and safety of AT2S to adrenergic vasopressors when used for perioperative hypotension in kidney transplant.

**Methods:**

This single-center, retrospective cohort study included adults with perioperative shock requiring AT2S or adrenergic agents as first-line vasopressors during kidney transplant. The primary outcome was the need for a second continuous infusion vasopressor agents beyond the first-line agent. Secondary outcomes assessed adverse events and early allograft outcomes.

**Results:**

Twenty patients receiving AT2S and 60 patients receiving adrenergic vasopressor agents were included. Intraoperatively, 1 of 20 patients (5%) in the AT2S group needed a second continuous vasopressor compared to 7 of 60 patients (11.7%) who needed a second continuous vasopressor in the adrenergic vasopressor group (*P* = 0.672). Postoperatively, 1 of 20 patients (5%) in the AT2S group compared to 12 of 60 patients (20%) in the adrenergic vasopressor group required a second vasopressor (*P* = 0.168). There were significantly fewer arrhythmias (1/20 [5%] vs. 17/60 [28.3%]), *P* = 0.03) and ischemic complications (0/20 [0%] vs. 11/20 [18.3%], *P* = 0.031) in patients who received AT2S. There were no differences in immediate, slow, or delayed graft function or in discharge, 1-month, and 3-month glomerular filtration rates (p > 0.05).

**Conclusion and Relevance:**

Both AT2S and adrenergic vasopressors are effective for perioperative hypotension in kidney transplant, with AT2S showing a lower incidence of arrhythmias and ischemic complications.

## Introduction

The inability to meet blood pressure (BP) goals in the perioperative period of kidney transplantation has been shown to carry significant consequences [1, 2]. Durations as short as 20 min of intraoperative mean arterial pressure (MAP) less than 55 mmHg have been associated delayed graft function (DGF), or even allograft loss. Though specific systolic blood pressure (SBP) and MAP goals in the perioperative period of kidney transplantation have not been formally declared, studies suggest allograft benefit when maintaining a SBP of ≥ 120 mmHg or a MAP of ≥ 95 mmHg [1, 3–5]. It is important to note that emerging evidence may suggest different goals than historical targets but thre precise goal surrounding kidney transplant surgery is still unknown (6).

In the transplanted denervated kidney, renal blood flow is dependent on systemic blood flow as hemodynamic autoregulation is impaired. This systemic blood flow is maintained via cardiac output (CO) and systemic vascular resistance (SVR) and in patients with adequate CO who remain hypotensive, blood flow is, therefore, dependent on increasing SVR with vasopressors [6–9]. However, evidence suggests that restoration of systemic BP alone is insufficient to ensure adequate renal perfusion as kidney perfusion depends on inflow and outflow pressures [10, 11]. The inflow pressure is often assumed to be similar to systemic BP, but the outflow (i.e., post-glomerular arteriole pressure) is often much lower than systemic BP [10]. Therefore, though vasopressors may improve systemic BP, the mechanism of vasoconstriction at the level of the kidney is vital. If an agent causes afferent arteriole vasoconstriction alone without any or minimal constriction of the efferent arteriole, this may worsen renal blood flow [11]. This is of particular concern during the reperfusion period of kidney transplant where activation of G-protein coupled receptors by endothelin, reduced nitric oxide production, and increased endothelial reactivity to vasoactive substances compound afferent arteriole vasoconstriction [12–14]. Renal microcirculatory dysfunctions have been observed and reported with the use of adrenergic vasopressors which raises concern about introducing renal risk in kidney transplant patients when using adrenergic vasopressors [15–17].

Clinically in kidney transplant, perioperative adrenergic vasopressor use has been associated with negative allograft outcomes, including decreased urine output, slower normalization of recipient serum creatinine (SCr), delayed graft function (DGF) in recipients, an increased rate of rejection [7, 9, 18, 19] and adverse events of tachycardias and the need for insulin infusions [7, 19]. Lastly, adrenergic vasopressors have been associated with increased length of stay (LOS) and increased mortality [8, 18, 19].

Ideally, in the setting of kidney transplant, a vasopressor would equally constrict both the afferent and efferent arteriole of the kidneys to maintain perfusion and intraglomerular pressure while avoiding ischemia and arrhythmic side effects. In 2017, the FDA approved pharmacologic angiotensin II (AT2S) for use in distributive shock [20]. Studies show that AT2S provides more balanced vasoconstriction within the renal vasculature and yields a net result of increased filtration fraction, which can help preserve kidney function [21] and as AT2S works through the non-adrenergic angiotensin II type 1 receptor, may decrease the risk of arrhythmias compared to catecholamine vasopressor agents like other non-catecholamines have demonstrated [22]. Though AT2S has been studied in other patient populations with distributive shock, there is minimal information about the use of AT2S in kidney transplant and few comparative research studies comparing AT2S to adrenergic vasopressors [6, 23, 24].

The aim of this study was to evaluate and compare AT2S to the previously available agents adrenergic vasopressors, as a first-line vasopressor in the management of perioperative hypotension in kidney transplant recipients. We hypothesized that AT2S, as compared to adrenergic vasopressor agents in the perioperative kidney transplant setting, would be similarly effective and have fewer adverse events when used as a first-line vasopressor agent.

## Patients and methods

This institutional review board (IRB) approved, single-center, retrospective before and after study compared patient outcomes between patients who received AT2S or adrenergic vasopressors as their first-line vasopressor for perioperative hypotension. AT2S was a new agent that was piloted for addition to formulary at our institution. This study compared the first 20 patients that received AT2S to a previous cohort of 60 patients receiving adrenergic vasopressors immediately before. Considering its retrospective nature, the Office for the Protection of Research Subjects (OPRS) issued a waiver of informed consent (Protocol: 2022–1122) in accordance with standards aligned with the Declaration of Helsinki.

Patients were included if they were > 18 years old, underwent a kidney transplant, had a pre-transplant ejection fraction (within 18 months of transplantation) of > 50%, and had intraoperative or postoperative hypotension requiring vasopressor support between 9/2020 and 5/2021. Patients were excluded if they were pregnant, prisoners, had a history of mesenteric ischemia, aortic dissection, abdominal aortic aneurysm, had an allergy to mannitol, had an absolute neutrophil count < 1000 cell/mm3 (within the past 18 months of transplantation), or had a diagnosis of Raynaud’s phenomenon, systemic sclerosis, or vasospastic disease.

Management of perioperative hypotension at our center followed a standardized protocol throughout the study period differing only in the choice of initial vasopressor. As the literature suggests achieving and maintaining an SBP of ≥ 120 mmHg in the perioperative period of kidney transplant [1, 4, 5, 18], the protocol defined hypotension as a sustained SBP below 120 mmHg, measured invasively via arterial line, throughout surgery and up to 24 h post-operatively. Hypovolemia was treated with fluid administration and significant surgical blood loss was treated with transfusion. Hypotension refractory to fluids led to initiation of vasoactive agents, either adrenergic agents or AT2S. If the first-line agents did not achieve hemodynamic goals, second and third agents could be added to achieve hemodynamic goals. For patients that received AT2S, the infusion was initiated at a rate of 20 ng/kg/min and titrated in increments of 5 ng/kg/min every 5 min, as needed. During the first 3 h of therapy, the maximum infusion rate of AT2S was 80 ng/kg/min. Patients that received adrenergic vasopressors could have received dopamine (DA), epinephrine (EPI), norepinephrine (NE), or phenylephrine (PE). Given the retrospective nature, the treatment team collectively determined the initial vasopressor choice and titrated each vasopressor according to institutional practices. All vasopressors were targeted to achieve and maintain a SBP goal of ≥ 120 mmHg without the need for vasopressor support.

The primary outcome was the need for any additional continuous vasopressor infusion (i.e., the need for > 1 vasopressor) beyond the first-line continuous infusion agent between groups that received AT2S or adrenergic agents first-line. Hemodynamic parameters were measured both intraoperatively and postoperatively.

The secondary outcomes were the safety and allograft outcomes in patients receiving AT2S or adrenergic vasopressors. Safety was tested by collecting the cumulative incidence of new-onset intra- and postoperative arrhythmias, digital, visceral, and/or other peripheral ischemia occurring during hospitalization for kidney transplant (as noted in chart documentation), or the need for insulin infusions while receiving the study drug. Arrhythmias were confirmed via electrocardiogram (EKG) flowsheet or note documentation and included atrial tachycardia (Atach), supraventricular tachycardia (SVT), atrial fibrillation (AF), atrial flutter (Aflutter), ventricular tachycardia (VT), and ventricular fibrillation (VF). Allograft function was evaluated by measuring the incidence of DGF, immediate graft function (IGF), slow graft function (SGF), and 1- and 3-month post-transplant estimated glomerular filtration rate (eGFR). DGF was defined as a requirement of renal replacement therapy by POD7 after transplantation. IGF was defined as > 50% decrease in SCr by POD7. SGF was defined as < 50% decrease in SCr by POD7.

All normally distributed continuous variables were summarized using means with standard deviations (SD), and all nonnormally distributed continuous variables and ordinal variables were summarized using medians and the interquartile range (IQR). Continuous outcomes were compared using a T-test if normally distributed or the Mann–Whitney if nonnormally distributed. Categorical outcomes were compared using a χ2 test or Fischer’s exact test. Our sample size was determined by the availability of data in our new electronic medical record and an a priori power calculation was not completed. Our institution changed electronic medical record systems in the fall of 2020 with AT2S being piloted for formulary addition starting in January 2021 leading us to utilize a sample size of convenience. A P-value of less than 0.05 was considered statistically significant.

## Results

Eighty patients were identified and met inclusion: 20 received AT2S and 60 received adrenergic vasopressors. Baseline demographics were similar except there were differences between groups regarding mean (SD) cold ischemic time (CIT) (14.7 (8.6) hours vs. 7.9 (5.6); P < 0.001), donor terminal SCr (1.79 (1.7) mg/dL vs. 1.11 (0.86) mg/dL; *P* = 0.027), and time on dialysis preoperatively (86.7 [53.3] months vs. 65.7 [36.1] months; *P* = 0.036) being statistically higher in the AT2S group (Table 1). Additionally, the baseline diastolic blood pressure (DBP) and baseline mean arterial pressure (MAP) were statistically lower (*P* = 0.05, *P* = 0.044, respectively) and heart rate (HR) statistically higher (P < 0.001) in the AT2S group (Table 2). Numerically, a greater number of patients receiving AT2S intraoperatively received continuous intravenous sedation with propofol (55% vs. 31.7%), but this did not reach statistical significance (*P* = 0.062; Table 1). There was a similar number of patients who received intravenous push propofol intraoperatively (Table 1).

**Table 1:** Baseline Characteristics

**Variable**	**AT2S **(*n*=20)	**Adrenergic** **Vasopressors**(*n*=60)	***p***-value
Age, years	56.3 (10.8)	53.1 (14.6)	0.369
Female Sex, n (%)	11 (55)	9 (45)	0.168
Race/ethnicity, n (%)			
-Black	6 (30)	10 (16.7)	0.197
-White	7 (35)	25 (41.7)	0.598
-Hispanic	5 (25)	22 (36.7)	0.339
Height, cm	167.8 (9.6)	169.1 (12.7)	0.67
Weight, kg	95.1 (24.2)	99.5 (29.3)	0.544
BMI, kg/m2	33.2 (6.8)	34.9 (10.9)	0.515
Dialysis preoperatively, n (%)	20 (100)	52 (86.7)	0.191
Time on dialysis, months	86.7 (53.3)	65.7 (36.1)	0.036
Pertinent pre-transplant comorbidities, n (%)			
-Hypertension (%)	18 (90)	56 (93.3)	0.624
-Atrial Fibrillation (%)	2(10)	5 (8.3)	0.672
-Coronary Artery Disease (%)	7(35)	15 (25.4)	0.215
-Diabetes Type II (%)	11(55)	15 (25.4)	0.518
Baseline Systolic BP, mmHg	145.2 (23.6)	157.3 (25.1)	0.063
Baseline Diastolic BP, mmHg	77.4 (13.4)	85.9 (17.3)	0.05
Baseline MAP, mmHg	98.7 (16)	108.1 (17.8)	0.044
Baseline heart rate, bpm	83 (17)	71.5 (9.9)	<0.001

*BMI* Body mass index, *BP *Blood pressure,* bpm* beats per minute, *cm* centimeter, *kg* kilogram, *MAP* Mean arterial pressure, *mmHg* millimeters of mercury, *SCr* Serum creatinine

All continuous data presented as means (standard deviation) unless otherwise noted

**Table 2:** Transplant Characteristics

**Variable**	**AT2S **(*n*=20)	**Adrenergic Vasopressors **(*n*=60)	***p***-value
Deceased donor transplant, n (%)	14 (70)	32 (54.2)	0.217
Donor terminal SCr, mg/dL	1.79 (1.7)	1.11 (0.86)	0.027
Kidney donor profile index, %	48.9 (27.8)	52 (26.7)	0.727
Donor age, years	45.4 (11.9)	41.2 (12.4)	0.199
Cold ischemic time, hours	14.7 (8.6)	7.9 (5.6)	< 0.001
Duration of surgery, hours	5.19 (1.9)	4.31 (2)	0.087
Thymoglobulin induction, n (%)	13 (65)	37 (61.7)	0.8
Basiliximab induction, n (%)	7 (35)	17 (28.3)	0.233
Tacrolimus maintenance immunosuppression, n (%)	20 (100)	56 (93.3)	0.567
Mycophenolate maintenance immunosuppression, n (%)	15 (75)	57 (95)	0.021
Intraoperative estimated blood loss, mL	278.3 (383.5)	189.3 (140)	0.196
Intraoperative continuous infusion propofol, n (%)	11 (55)	19 (31.7)	0.062
Intravenous push propofol, n (%)	20 (100)	58 (96.7)	0.408
-Dose of propofol, mg	133.8 (61.9)	131.2 (53.7)	0.858

AT2S = synthetic angiotensin II

All continuous data presented as means (standard deviation) unless otherwise noted

**Fig. 1:**
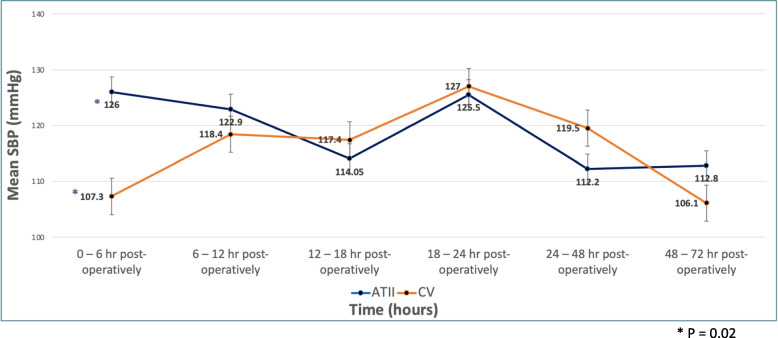
Average Systolic Blood Pressures (SBP) vs. Time. This chart compares the average SBP between groups. The dark blue line denotes the angiotensin II group (ATII) and the light orange line denotes the catecholamine vasopressors group (CV). P values were statistically similar except during the 0–6 h postoperative period

Dopamine was the primary adrenergic agent for intraoperative usage (85.7%) and postoperative use (51.2%) in the adrenergic group. Both groups had similar hemodynamic outcomes (Table 3). Eighty percent of patients in the AT2S group required intraoperative continuous vasopressors compared to 70% in the adrenergic vasopressor group (*P* = 0.386). One out of 20 patients (5%) in the intraoperative setting needed a second continuous vasopressor beyond AT2S compared to 7 out of 60 patients (11.7%) who needed a second continuous vasopressor beyond their first-line adrenergic vasopressor (*P* = 0.672). Postoperatively, a similar rate of patients required vasopressors between the groups (12/20 [60%] vs. 41/60 [68.3%], *P* = 0.495). In this setting, one (5%) patient in the AT2S group required an second vasopressor compared to 12 (20%) in the adrenergic vasopressor group (*P* = 0.168). Intraoperative duration of vasopressors was similar between groups (Table 2) but postoperative usage of AT2S was statistically longer (49.8 [37.4] hours vs. 25 [30.4] hours, *P* = 0.019). Patients receiving AT2S had a statistically higher SBP in the early (0 – 6 h) response period after starting vasopressors (126 [15.8] mmHg vs. 107.3 [29.5] mmHg, *P* = 0.02) but there were no statistical differences at all other time points (P > 0.05) (Fig. 1).

**Table 3:** Hemodynamic Outcomes (Primary)

**Variable**	**AT2S **(*n*=20)	**Adrenergic** **Vasopressors (n=60)**	**p-value**
Patients with intraoperative vasopressors, n (%)	16 (80)	42 (70)	0.386
Number of vasopressors intraoperatively, n (%)			
-0	4 (20)	18 (30)	0.386
-1	15 (75)	35 (58.3)	0.182
-2	1 (5)	7 (11.7)	0.389
Duration of vasopressors intraoperatively, hours	2.5 (2.2)	2.1 (1.7)	0.527
First continuous vasopressor intraoperatively, n (%)			
-Angiotensin II	15 (100)	0	
-Dopamine	0	36 (85.7)	< 0.001
-Epinephrine	0	1 (2.4)	
-Norepinephrine	0	0	
-Phenylephrine	0	5 (11.9)	
Intra-operative norepinephrine equivalents ^T^ (mcg/kg/min)	0.046 (0.04)	0.085 (0.12)	0.203
Push dose vasopressors intraoperatively, n (%)	16 (80)	41 (68.3)	0.318
Patients with blood products administered intraoperatively, n (%)	11 (55)	20 (33.3)	0.085
Crystalloid volume intraoperatively, mL	3498 (1591)	3465 (1588)	0.937
Patients with postoperative vasopressors, n (%)	12 (60)	41 (68.3)	0.495
Number of vasopressors postoperatively, n (%)			
-0	6 (30)	17 (28.3)	0.887
-1	11 (55)	29 (48.3)	0.606
-2	1 (5)	12 (20)	0.115
Duration of vasopressors postoperatively, hours	49.8 (37.4)	25 (30.4)	0.019
Patients with blood products administered postoperatively, n (%)	1 (5)	4 (6.7)	0.818
First continuous vasopressor postoperatively, n (%)			
-Angiotensin II	12 (100)	0	
-Dopamine	0	21 (51.2)	< 0.001
-Epinephrine	0	1 (2.4)	
-Norepinephrine	0	1 (2.4)	
-Phenylephrine	0	18 (43.9)	
Post-operative norepinephrine equivalents (mcg/kg/min)*	0.05 (0.02)	0.12 (0.14)	0.084

AT2S = synthetic angiotensin II

All continuous data presented as means (standard deviation) unless otherwise noted

*Calculated from [25]: Kotani, Y., Di Gioia, A., Landoni, G. *et al.* An updated “norepinephrine equivalent” score in intensive care as a marker of shock severity. Crit Care 27, 29 (2023). https://doi.org/10.1186/s13054-023-04322-y

When comparing safety outcomes, the AT2S group had significantly fewer arrhythmias compared to the adrenergic vasopressors group (1/20 [5%] vs. 17/60 [28.3%], *P* = 0.03). Additionally, patients developed fewer documented digital, peripheral, or visceral ischemic complications (0/20 [0%] vs. 11/60 [18.3%], *P* = 0.031). The development of ischemic complications was statistically correlated with both using an adrenergic agent (*P* = 0.038) and the average postoperative norepinephrine dosing equivalents (*P* = 0.015). There were no differences in the need for insulin infusions between groups (Table 4). There were zero thrombotic events (0/20 [0%] vs. 0/60 [0%], *P* = not applicable) documented in either group. All patients in both groups survived until hospital discharge (20/20 [100%] vs. 60/60 [100%]; *P* = not applicable).

**Table 4:** Safety Outcomes

**Variable**	**AT2S (n=20)**	**Adrenergic** **Vasopressors (n=60)**	**p-value**
Intra- or postoperative arrhythmia, n (%)	1 (5)	17 (28.3)	0.03
Digital or other peripheral/visceral ischemia, n (%)	0	11 (18.3)	0.031
Need for insulin infusion, n (%)	5 (25)	16 (26.7)	0.883
Thrombotic events, n (%)	0 (0)	0 (0)	n/a

*AT2S* synthetic angiotensin II, *eGFR* estimated glomerular filtration rate, *n/a* not applicable, *SCr* Serum creatinine

Despite having a statistically higher first postoperative SCr (7.84 [3.32] mg/dL vs. 3.32 [1.84] mg/dL, *P* = 0.02), function and laboratory allograft outcomes did not differ significantly between groups (Table 5). The incidence of DGF (2/20 [10%] vs. 18/60 [30%], *P* = 0.083, IGF (14/20 [70%] vs. 37/60 [61.7%], *P* = 0.597), SGF (4/20 [20%] vs. 5/20 [8.3%], *P* = 0.217), 1-month post-transplant eGFR (44.9 [24.3] mL/min/1.73m2 vs. 48.5 [17.2] mL/min/1.73m2, *P* = 0.6), and 3-month post-transplant eGFR (46.7 [19.9] mL/min/1.73m2 vs. 51.6 [19.4] mL/min/1.73m2, *P* = 0.46), were statistically similar between AT2S and adrenergic vasopressors groups, respectively.

**Table 5:** Allograft Outcomes

**Variable**	**AT2S (n=20)**	**Adrenergic** **Vasopressors (n=60)**	**p-value**
First postoperative SCr, mg/dL	7.84 (3.32)	3.32 (1.84)	0.02
Discharge SCr, mg/dL	3.28 (2.54)	3.02 (2)	0.641
Discharge eGFR, mL/min/1.73m2	22.6 (26.9)	36.3 (24.2)	0.677
Immediate Graft Function, n (%)	14 (70)	37 (61.7)	0.597
Delayed Graft Function, n (%)	2 (10)	18 (30)	0.083
Slow Graft Function, n (%)	4 (20)	5 (8.3)	0.217
Poor Graft function, n (%)	6 (30)	23 (38.3)	0.502

*AT2S *synthetic angiotensin II, *eGFR* estimated glomerular filtration rate, *n/a* not applicable, *SCr* Serum creatinine

All continuous data presented as means (standard deviation) unless otherwise noted

## Discussion

In our single-center, retrospective cohort analysis, there were no statistical differences in the need for a second vasopressor among patients who received AT2S compared to those who received adrenergic vasopressors. Furthermore, patients who received AT2S exhibited fewer adverse events of arrhythmias and ischemic complications, and there were no discernible differences in allograft outcomes. Given the growing interest in the broader transplant community for utilizing this agent [26–29] and the desire to clarify the role of this agent in other forms of distributive shock [24] the analysis results are pivotal for several reasons.

First, maintaining hemodynamic stability is paramount in the kidney transplant population, as failure to achieve BP goals in the perioperative period is known to adversely impact kidney outcomes [1, 2]. While adrenergic vasopressors have historically facilitated BP goal attainment, their association with negative outcomes in the kidney transplant population poses significant challenges from both renal and non-renal perspectives [6–9, 19]. In light of this evidence, agents with alternative mechanisms, such as vasopressin, methylene blue, hydroxocobalamin, and AT2S, may be preferred. Vasopressin, which exerts its hemodynamic effects via the AVPR1a receptors, has not demonstrated significant impacts on renal outcomes [30–32]. Similarly, data regarding the use of methylene blue for vasoplegic syndrome in transplantation is limited to case reports, with its efficacy and safety profile still inadequately elucidated [33, 34]. While meta-analysis in the septic shock and cardiac surgery population is promising [35], the use in the post kidney transplant population remains uncertain. Hydroxocobalamin, primarily utilized for catecholamine-refractory vasoplegia in transplant settings, has also not been extensively investigated as a first-line agent [36]. Consequently, AT2S emerges as a potentially valid alternative first-line agent in this context.

Second, our findings suggest comparable effectiveness and illuminate a preferential benefit with early AT2S administration. In our study, patients who received AT2S demonstrated a higher average SBP within the immediate (0–6 h) postoperative period, suggesting a more robust initial response to AT2S compared to adrenergic vasopressors. This finding aligns with observations in other shock populations as well. The ATHOS-3 Phase 3 clinical trial showed a rapid hemodynamic response as patients receiving AT2S achieved their MAP goal with a median duration of 5 min, an effect that was sustained throughout the study period [23]. The observed hemodynamic responsiveness to AT2S could in part be due to the benefits of mimicking the natural physiologic response to hypotension by correcting renin–angiotensin–aldosterone system (RAAS) derangements [23]. The RAAS is an essential hormonal regulator responsible for maintenance of BP via the actions of AT2S. In transplant surgical populations like the one investigated in this analysis, factors like brain death in donors, cold ischemic injury, and warm reperfusion trigger an outpouring of inflammatory cytokines leading to RAAS disruptions and subsequent hypotension [12–14, 37, 38]. Thus, prompt administration of exogenous AT2S could aid in correcting RAAS derangements and swiftly restore and maintain BP.

Third, we observed a higher incidence of postoperative adverse events, including tachyarrhythmias and ischemic complications, among patients who received adrenergic vasopressors. Studies show that arrhythmias (e.g., AF) is a prevalent issue within the kidney transplant population [39, 40]. Lentine, et al. reported that AF affected up to 7% of renal allograft recipients by the third year post-transplantation, with the highest rate of diagnosis occurring during the perioperative phase of kidney transplant [40]. This aligns with existing literature linking surgical stressors, such as excess catecholamine production and autonomic imbalances, with cardiac arrhythmias [41]. Hence, the addition of catecholamine vasopressors could potentially compound the risk of AF. Notably, Gordon et al. observed a higher incidence of life-threatening arrhythmias among septic shock patients receiving catecholamine vasopressors compared to non-catecholamine vasopressors recipients [32]. Similarly, the ATHOS-3 trial found that patients who received catecholamine vasopressors had a higher incidence of cardiac disorders compared to those who received AT2S [23]. We observed that dopamine was the most commonly administered vasopressor during the perioperative period. This agent is notorious for being associated with arrhythmias [42] and is being suggested less frequently for patients with shock [43]. It would be intriguing to perform a similar comparison in patients receiving less arrhythmic adrenergic agents such as NE. Regardless, this agent is still being utilized in critically ill patients [44]. AF following kidney transplant has been independently associated with increased mortality and death-censored graft failure [40], underscoring the importance of our findings, which indicate a lower incidence of tachyarrhythmias among patients who received AT2S. This highlights the necessity for further investigation into the implications of our results.

Lastly, we observed comparable rates of early allograft dysfunction between groups and similar long-term graft survival. This observation carries clinical significance, particularly as we relied on functional markers rather than laboratory values alone. Studies suggest that the odds of DGF increase by 4% for every 1-h increase in CIT [45]. Likewise, donor terminal SCr has been significantly associated with DGF, with odds exceeding 60% for each 1 mg/dL increase in SCr [45]. Therefore, considering the significantly prolonged CIT and higher donor SCr levels in the AT2S group, one would anticipate the AT2S group to have higher odds of DGF. This would be anticipated, especially with the significant differences in first postoperative SCr. However, contrary to expectations, the observed rates of DGF were similar between groups and trended toward favoring AT2S, and neither early nor long-term graft survival was affected. Though hypothetical, our data could suggest that AT2S may have mitigated some of the potential risks of DGF that would have otherwise been observed. Mechanistically, the more balanced vasoconstriction of AT2S within the renal vasculature results in a net increase in filtration fraction, which ultimately could help preserve allograft and kidney function [21]. This has been confirmed with animal and human clinical studies in septic shock patients, who face the highest known risk of developing AKI [46, 47]. However, given our study's small sample size and retrospective nature, our findings warrant further investigation. Nonetheless, our results may offer providers another avenue for managing perioperative hypotension in kidney transplant patients.

There are several limitations to our study, primarily due to the design of our study. First, given the retrospective study design of this evaluation, important data points may be missing, hard to interpret, or difficult to capture retrospectively. This may limit the ability to compare infrequent side effects between groups, such as arrhythmias or peripheral ischemia. We attempted to objectively define these variables as able in a retrospective analysis and use commonly reported mechanisms to investigate these adverse effects. Similarly, though we only included arrhythmias deemed clinically consequential (i.e., Atach, SVT, AF, Aflutter, VT, and VF), we did not collect the duration of perioperative arrhythmias that each patient experienced, or the clinical outcomes associated with those arrhythmias, which remains a limitation [39, 40, 48]. Secondly, subtle patient differences between cohorts may have influenced the observed results. This would include subtle differences such as the timing of the last dialysis session in relation to the transplant surgery which could change the comparison of immediate allograft outcomes. Additionally, we were not able to determine the exact clinical consequences that led to vasopressor initiation or utilization. However, as every patient received vasopressors according to our standardized protocol with the exception of agent choice, we feel these differences in events preceding the use of vasopressors may be clinically inconsequential. Further, we did not have data collected on volatile anesthetics or the doses of intraoperative propofol. Given the relatively short time period of patients included, it is unlikely that our intraoperative practices would be very different across the study cohort. Third, the external validity of our findings may be limited due to the single-center nature of our study. This becomes relevant when considering institutions that use different adrenergic vasopressor agents, as their proarrhythmic properties are known to differ [49]. Additionally, the demographic make-up is likely different at other institutions which may lead to different vasopressor durations perioperatively limiting generalizability [19, 50]. Nevertheless, we included a racially diverse patient population with high inherent risks for early allograft dysfunction, which we believe is a strength given the findings of our study. Further, ideally, we would have the ability to collect renin levels to assess the RAAS profile of patients, this may assist in providing additional clarity surrounding patients that may benefit from AT2S usage [51–53]. Lastly, the small sample size being one of convenience, we likely had inadequate power to detect small differences in our primary outcome. Consequently, further investigation with larger sample sizes is warranted to confirm our results.

## Conclusion and relevance

In conclusion, AT2S and adrenergic vasopressors were both adequate when used for perioperative hypotension surrounding kidney transplant, but patients receiving AT2S experienced a lower incidence of adverse events. Larger trials are warranted to assess this further as the literature is currently lacking in this patient space.

## Data Availability

GU and SB conceptualized the study. EB, HN, and JB reviewed the study protocol and provided guidance for its execution. GU and SB performed data collection and analysis. GU and SB composed the drafts of the manuscript, and all authors reviewed the final version of the manuscript.
